# Analyzing the Impact of Storage Shortage on Data Availability in Decentralized Online Social Networks

**DOI:** 10.1155/2014/826145

**Published:** 2014-05-04

**Authors:** Songling Fu, Ligang He, Xiangke Liao, Kenli Li, Chenlin Huang

**Affiliations:** ^1^School of Computer Science, National University of Defense Technology, Changsha 410073, China; ^2^School of Information Science and Engineering, Hunan University, Changsha 410082, China; ^3^Department of Computer Science, University of Warwick, Coventry CV4 7AL, UK

## Abstract

Maintaining data availability is one of the biggest challenges in decentralized online social networks (DOSNs). The existing work often assumes that the friends of a user can always contribute to the sufficient storage capacity to store all data. However, this assumption is not always true in today's online social networks (OSNs) due to the fact that nowadays the users often use the smart mobile devices to access the OSNs. The limitation of the storage capacity in mobile devices may jeopardize the data availability. Therefore, it is desired to know the relation between the storage capacity contributed by the OSN users and the level of data availability that the OSNs can achieve. This paper addresses this issue. In this paper, the data availability model over storage capacity is established. Further, a novel method is proposed to predict the data availability on the fly. Extensive simulation experiments have been conducted to evaluate the effectiveness of the data availability model and the on-the-fly prediction.

## 1. Introduction

In the last decade, online social networks (OSNs), such as Facebook [1], Twitter, and Sina Weibo [2], have gained extreme popularity with more than a billion users worldwide. OSNs allow a user to publish the data to all his friends in his friend circle.

Currently, the OSN platforms are typically centralized, where the users store their data in the centralized servers deployed by the OSN service providers. The service providers can utilize and analyze these data to know the users' private information, such as interest and personal affairs, and in the worst case may sell this information to the third party. Therefore, the current centralized online social networks (COSNs) have raised the serious concerns in privacy [3–6].

In order to address the data privacy issue, the decentralized online social networks (DOSNs) have been proposed recently [7–11]. Although the DOSN products [12] are not as popular and mature as the OSN products [1], DOSN is indeed under active research and development [13–17]. In DOSNs, in order to protect the data privacy the centralized servers are bypassed and the data published by a user are stored and disseminated only among the friend circle of the user [9, 10]. Although DOSNs can help protect the data privacy, maintaining data availability becomes a big challenge. This is because if a friend of the user is offline, the data stored in the friend cannot be accessed by other friends.

In order to achieve good data availability in DOSN, the data replication approach has been widely used. In this approach, a certain number of data replicas are created for each data item published by a user and these data replicas are stored in the user's friend circle. By doing so, if a friend is offline, the data in this offline friend node can be accessed through the replicated data stored in other friend nodes.

In the existing data replication work in DOSN, it is typically assumed that the friends of a user are always capable of contributing sufficient storage capacity to store all the published data [9, 14, 18]. This assumption is not ideal, especially in the current modern times. Nowadays, the users often use smart mobile devices, such as smart phones, to access the OSN services. The resources in the mobile devices are much more limited than the desktop computers used in the “old fashioned” style of accessing OSNs. Moreover, the number of the friends in a friend circle is limited (typically less than 200) [19]. Therefore, it is desired to know what level of data availability can be achieved given the total storage capacity contributed by the friend circle. However, the existing work in DOSN has not yet conducted quantitative research in this aspect.

This paper aims to address the above issue and build a quantitative model to capture the relation between the total storage capacity contributed by the friends and the level of data availability in the DOSN.

The reason why we investigate the relation between the total storage capacity and data availability is because a data item is regarded as being available as long as it is stored in the online friend nodes in the DOSN, no matter which online friends the data replicas are stored in. The location of the data replicas does not directly affect the data availability but mainly imposes the impact in the following two aspects.

- Data accessing performance: due to, for example, the bandwidth and latency of the friends where the data are stored, other friends who are accessing the data may experience different performance.
- The data maintenance overhead: when a friend goes offline, the data replicas on the friend have to be generated on other online friends. Various attributes of the friend, such as the storage capacity contributed by this friend, bandwidth, and latency, have impact. For example, if a friend offers the big storage capacity, then potentially more data have to be generated in other friends when this friend goes offline.

How to optimize data accessing performance and reduce data maintenance overhead is the work of the underlying data replication and placement strategies. This work is situated at the level of maintaining data availability. This is why this work mainly concerns the total storage size provided by the friends collectively. Following on from this work, we plan to work down the management levels in DOSN and develop the placement strategies for data replicas among the friends in DOSN.

In order to build the data availability model, we need to have deep understandings of the DOSN properties that are related to data availability. In this paper, we analyze these relevant properties and establish the probabilistic models for them. Further, the models for the individual properties are integrated to construct the data availability models. Further, a novel method is proposed to predict the level of data availability on the fly.

Using the data availability model developed in this paper, the DOSN designers can determine the average size of the storage pool that each friend should contribute for the published data, given the level of data availability that the DOSN desires to achieve. Moreover, in DOSN, the friends become online and offline dynamically; the data availability will drop when the number of online friends decreases. The on-the-fly prediction method can be used to conduct the real-time prediction for the level of data availability in the near future. The quantitative prediction results produced by the model can greatly help the data replication and storage policies make judicious decisions on the fly.

The rest of this paper is organized as follows.  Section 2 discusses related work about analyses of OSN properties, the existing DOSN approaches, and data availability work.  Section 3 states the problem which we try to address.  Section 4 presents the data availability model over storage capacity.  Section 5 presents the on-the-fly prediction model.  Section 6 shows some case study.  Section 7 conducts extensive experiments to verify our models and analyzes experimental results. Finally, we make conclusions.

## 2. Related Work

This section discusses the related work mainly in the following three aspects: (i) the existing work of analyzing the OSN properties, including both the characterizations of OSN networks and the analyses of user behaviors (Section 2.1), (ii) the existing research on DOSN, that is, the alternative approaches to decentralizing the OSNs (Section 2.2), and (iii) the existing studies on data availability in DOSN (Section 2.3). Moreover, this section also discusses the existing work in achieving data availability in grids and clouds (Section 2.4).

### 2.1. Analyses of the OSN Properties

#### 2.1.1. Characterizations of OSN Networks

Some studies use the graphs to represent the OSN networks and investigate the graph structures of OSN, such as degree distribution, network diameter, and clustering property. They conduct the analyses through the crawled data gathered from popular OSN sites such as Facebook, Twitter, MySpace, Flickr, YouTube, LiveJournal, Cyworld, and orkut [13, 19–22]. It has been found that (i) OSNs manifest power-law, small-world, and scale-free properties; (ii) the social network is nearly fully connected; (iii) the neighborhoods of the users in the social graph contain the surprisingly dense structure, while the graph is sparse as a whole; (iv) most users have a moderate number of friends (less than 200). The findings about the number of friends will be used to design the simulation experiments in this paper.

#### 2.1.2. Analyses of User Behaviours

The work in [23–27] studied the patterns of the user behaviors through the crawled or clickstream data. Jin et al. [23] conducted a comprehensive review about the user behavior in OSNs from several perspectives, including social connectivity and interaction among users, traffic activity, and the characteristics in mobile environments. Benevenuto et al. [24] collected the clickstream data over 12 days to study the characteristics of OSN sessions, including the accessing frequency, session durations, and total time spent on OSNs. Schneider et al. [25] focused on feature popularity, session characteristics, and the dynamics in the OSN sessions. Kwon and Wen [26] empirically examined how the individual characteristics affect the actual user acceptance of social network services. Yan et al. [27] studied the human behavior using the data obtained from the “Sina Microblog,” which is one of the most popular OSN sites in China. They found that the human activity patterns are heterogeneous and bursty and often follow the power-law distribution.

Since the existing research has revealed the dynamic characteristics about user behaviors, such as the distributions of online and offline durations, these will be used as the known parameters when we derive the data availability model and the on-the-fly prediction in this paper.

### 2.2. DOSN

To address the data privacy problem in COSNs, several decentralized approaches have been proposed [7–11]. Buchegger et al. [7] proposed a decentralized, peer-to-peer approach coupled with encryption. Yeung et al. [8] adopted a decentralized approach by using the URIs as the identifiers throughout, which can provide the same (or even higher) level of user interaction as with many of the current popular OSN sties. Tandukar and Vassileva [9] also proposed a decentralized OSN. With this approach, users can maintain the control over their data to protect their data privacy and forward the social data selectively to reduce the irrelevant data among the users. None of these approaches only stores the data published by a user in his friend circle.

There is another type of DOSNs [10, 11], known as friend-to-friend storage systems, which focus on providing the data storage services for all participants. Li and Dabek [10] argued that a node should choose its neighbors where the data are stored based on existing social relationships instead of randomly. Sharma et al. [11] find that the limitation of storing data only on friends has a marked impact on the data availability. They showed that the problem of obtaining maximal availability while minimizing redundancy is NP complete and proposed greedy data placement heuristics to improve the data availability. Our data availability model and the on-the-fly prediction can be integrated into these existing DOSNs; for example, the quantitative results produced by our models can be used to help make the data replication and/or data storage decisions.

### 2.3. Data Availability in DOSN

Because of the requirement of protecting data privacy, the data published by a user are only stored in his friend circle in the DOSN. Consequently, data availability is one of the biggest challenges in DOSNs. The existing work in improving data availability mainly focuses on designing smart data replication and data storage policies.

Shakimov et al. [28] propose three schemes for storing the data in DOSNs: the cloud-based scheme, the desktop-based scheme, and the hybrid scheme combining the above two. In the cloud-based scheme, the data will be stored in the cloud servers. In the desktop-based scheme, two mechanisms may be used: (i) the data replicas are encrypted when they are stored in potentially untrusted hosts; (ii) the users take advantage of the trust embedded in the social network to store the data replicas on trustworthy friends. The drawbacks of these mechanisms come from the complexity and overhead in the encryption key or trust management.

The approach proposed by Koll et al. [18] exchanges the recommendations among the socially related nodes in order to effectively distribute a user's data replicas among the eligible nodes carefully selected in the OSN.

In the approach developed by Olteanu and Pierre [14], the preferences are given to the nodes when it comes to selecting the nodes for storing the data (and their replicas) published by a user [14]. The online friends of the user have the highest priority. When all friends are offline, the data are then stored in the nodes which are not in the user's friend circle.

Buchegger et al. designed a two-tiered DOSN architecture (PeerSoN) [7]. One tier serves as a look-up service which is implemented by OpenDHT. The second tier consists of the peers and contains the user data. When a user is offline, all his data will be stored across the whole network.

Cutillo et al. [29] propose a P2P-based DOSN (Safebook), in which each node is accessible through the so-called shells. The profile data is mirrored and stored in a subset of a node's direct contacts, which form the so-called innermost shell. The data retrieval requires traversing the shells along a path of the nodes that are online and are friends with each other.

Tegeler et al. [30] propose an approach called Gemstone. Gemstone protects the user's privacy by encrypting all data using ABE and stores the user's data in the so-called data holding agents (DHAs). If a DHA itself is offline, the data have to be passed to the DHAs of this offline DHA.

All the above existing work about data availability focuses on how to store the data replicas so that they are still accessible when the users or certain friends of the users are offline. They all implicitly assume that the friends are always able to contribute the adequate storage capacities to store the replicated data.

### 2.4. Data Availability in Grids and Clouds

We also studied the existing work in achieving data availability in grids and clouds. Amjad et al. [31] surveyed the dynamic replication strategies for improving data availability in data grids. Kossmann et al. [32] proposed a modular cloud storage system. Zeng et al. [33] studied the cloud storage architecture and then pointed out the key techniques.

However, we found that the focuses and the considerations in achieving data availability in grids and clouds are quite different from those in DOSN. One of the biggest differences is that the data replication mechanisms in grids or clouds do not treat the total storage capacity as a limitation, although some studies considered the case where the storage capacity of individual nodes in a grid system is limited. Namely, these studies all explicitly or implicitly assume that the total storage space in grids or clouds is always sufficient to store the data replicas. This assumption is reasonable for grids and clouds because of the scale of such systems. However, it is not always true for DOSN due to the aforementioned facts that (1) smart mobile devices, whose storage capacity is limited, are often used in DOSN and (2) the number of friends in a friend circle is also limited.

## 3. Problem Statement

**Figure 1:**
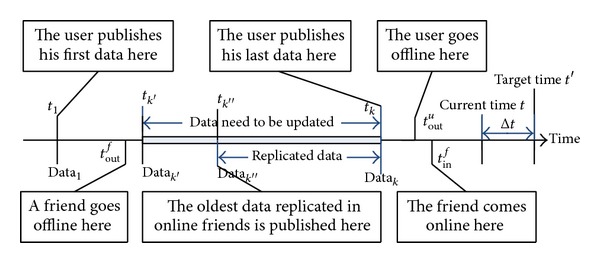
The illustration of the data availability problem.

Figure 1 illustrates the data availability problem. In Figure 1, the user publishes the data at a series of time points along the time line. Assume *t*_1_ is the first time point when he publishes the data, Data_1_, after he comes online, and *t*_*k*_ is the last time point the user publishes the data, Data_*k*_, before he goes offline at the time point *t*_out_^*u*^. Now let us consider one of the friends in the user's friend circle. Assume that the friend goes offline at time point *t*_out_^*f*^ just before the user publishes Data_*k*′_ (and after the user publishes Data_*k*′−1_) and then comes online at time point *t*_in_^*f*^ after the user goes offline. Therefore, Data_*k*′_ to Data_*k*_ are the data that the friend missed when he is offline and consequently need to be updated when he comes online. Since the user is already offline, the friend can only update the missed data from other online friends where the data replicas are stored. Note that if the friend comes online before the user goes offline, the friend can update all missed data from the user directly. Therefore, data availability is not a problem under this circumstance.

When a friend comes online, assume that the total amount of the data that the friend tries to update is *D*_update_. Out of *D*_update_, the amount of data that are stored in online friends of the user is *D*_stored_. The level of data availability (denoted by DA) is defined as

(1) $$\begin{array}{c} \text{DA}=\frac{D_{\text{stored}}}{D_{\text{update}}}. \end{array}$$

The data replication frameworks typically work in the following way [10, 18, 34]. When the user publishes a data item, a certain number of data replicas are created and stored in the storage pools of the selected friends of the user. When a friend goes offline the data replicas which are stored in this friend will be recreated and stored on other online friends to maintain fixed number of data replicas for each data item. If the size of the storage pools is unlimited, the new data will just be added to the friend's storage pool. If the storage pool is limited and the pool is already full, the oldest data in the storage pool will be replaced with the new data. Therefore, the size of the storage pool will determine what period of data is stored in the pool, which affects the data availability of the DOSN. Consider Figure 1 again; for example, if the storage pool in the friends is limited and can only store the data published from *t*_*k*_ back to *t*_*k*′′_, then the data earlier than *t*_*k*′′_ are not available when the friend comes online at *t*_in_^*f*^.

One aim of this paper is to establish the data availability model to capture the relation between the level of data availability and the total size of the storage pools contributed by the friends. This is presented in Section 4.

Now consider a time point *t*′ after the current time *t*. The other aim of this paper is to predict the level of data availability at *t*′ on the fly, which is presented in Section 5. This prediction is very useful for the data replication or storage policies to make judicious decisions dynamically.

The notations that are used in the derivations of the data availability models are introduced as Table 1.

## 4. The Data Availability Model over Storage Capacity

As discussed in Section 3, the total size of the storage pool contributed by a user's friends (denoted by SS) can determine the period of the published data stored in the storage pool. *t*_*tl*_ denotes the publishing time of the oldest data stored in the storage pool (i.e., *t*_*k*′′_ in Figure 1), and *t*_out_^*u*^ denotes the time when the user goes offline. Then [*t*_*tl*_, *t*_out_^*u*^] is the period of the published data stored in the storage pool. This section first determines *t*_*tl*_ (Section 4.1) and then presents the method of establishing the relation between SS and the DA of the data published by the user (Section 4.2).

### 4.1. Calculating *t*_*tl*_

In order to determine *t*_*tl*_, the size of the data published by the user has to be calculated first. *X*(*t*_pu_) denotes the number of times that the user publishes the data in the time duration *t*_pu_. *X*(*t*_pu_) is a discrete random variable. *P*_pu_(*x*(*t*_pu_)) denotes the probability density function (pdf) of *X*(*t*_pu_). *a* denotes the average size of the data published by the user each time. *S*(*t*_pu_) denotes the total size of the data published by the user in *t*_pu_. Clearly, *S*(*t*_pu_) = *aX*(*t*_pu_). Therefore, the pdf of *S*(*t*_pu_), denoted by *S*_pu_(*s*(*t*_pu_)), can be determined by (2) and the expectation of *s*(*t*_pu_) can be calculated by (3) as follows:

(2) $$\begin{array}{c} S_{\text{pu}}\left(s\left(t_{\text{pu}}\right)\right)=a\cdot P_{\text{pu}}\left(x\left(t_{\text{pu}}\right)\right), \end{array}$$

(3) $$\begin{array}{c} E\left[S\left(t_{\text{pu}}\right)\right]=a\cdot E\left[X\left(t_{\text{pu}}\right)\right]=a\cdot \overset{+\infty }{\underset{x=1}{\sum }}x\cdot P_{\text{pu}}\left(x\left(t_{\text{pu}}\right)\right). \end{array}$$

The publishing time of the oldest data stored in the storage pool, *t*_*tl*_, can be calculated using (4) given SS, where *k* is the replication degree in the OSN, that is, the number of replicas created for each data item. Consider

(4) $$\begin{array}{c} E\left[S\left(t_{\text{out}}^{u}-t_{tl}\right)\right]\cdot k=\text{SS}. \end{array}$$

### 4.2. Establishing the Relation between DA and SS

When a friend comes online at *t*_in_^*f*^ (as in Figure 1) and his last logout time (denoted by *t*_out_^*f*^) is no earlier than *t*_*tl*_, the friend can update all the data missed during his offline duration from other online friends. Namely, DA for a friend coming online at *t*_in_^*f*^, denoted by DA(*t*_in_^*f*^, *t*_out_^*f*^), is 100% in this case. When *t*_out_^*f*^ is earlier than *t*_*tl*_, the data published in [*t*_out_^*f*^, *t*_*tl*_] are not available to the friend. Therefore, DA in this case equals the proportion of the data that are published in [*t*_*tl*_, *t*_out_^*u*^] to those in [*t*_out_^*f*^, *t*_out_^*u*^]. In summary, DA(*t*_in_^*f*^, *t*_out_^*f*^) can be calculated using

(5) $$\begin{array}{c} \text{DA}\left(t_{\text{in}}^{f},t_{\text{out}}^{f}\right)=\{\begin{array}{cc} 100\% & t_{\text{out}}^{f}\geq t_{tl}\\ \frac{E\left[S\left(t_{\text{out}}^{u}-t_{tl}\right)\right]}{E\left[S\left({t_{\text{out}}^{u}-t}_{\text{out}}^{f}\right)\right]}\cdot 100\% & t_{\text{out}}^{f}<t_{tl}. \end{array} \end{array}$$

*t*_*off*⁡_ denotes the time duration of a friend being offline continuously. *f*_*off*⁡_(*t*_*off*⁡_) denotes the pdf of *t*_*off*⁡_. The probability that a friend went offline at *t*_out_^*f*^ and then comes online at *t*_in_^*f*^ is *f*_*off*⁡_(*t*_in_^*f*^ − *t*_out_^*f*^)*dt*_out_^*f*^ and the corresponding DA(*t*_in_^*f*^, *t*_out_^*f*^) is obtained by (5). Then, DA at time point *t*_in_^*f*^ can be expressed by

(6) $$\begin{array}{c} \overset{0}{\underset{t_{\text{out}}^{u}}{\int }}f_{\mathrm{off}}\left(t_{\text{in}}^{f}-t_{\text{out}}^{f}\right)\cdot \text{DA}\left(t_{\text{in}}^{f},t_{\text{out}}^{f}\right)dt_{\text{out}}^{f}. \end{array}$$

DA_[*t*_out_^*u*^,  *h*]_ denotes the expectation of DA over the time duration between *t*_out_^*u*^ and *t*_in_^*f*^, where *h* is the duration between the user's two consecutive logins (the work in [25, 35, 36] has presented the method to obtain the value of *h*). DA_[*t*_out_^*u*^,  *h*]_ can be calculated by (7), where *f*_at_(*t*_in_^*f*^) is the probability density function that a friend comes online at time *t*_in_^*f*^:

(7) DA[toutu,  h]=∫toutuhfat(tinf) ·∫toutu0foff⁡(tinf−toutf)DA(tinf,toutf)dtoutfdtinf.

DA_[0, *t*_out_^*u*^]_ denotes the expectation of DA over the time duration between 0 and *t*_out_^*u*^. Since the user is online between 0 and *t*_out_^*u*^, DA is 100% over the time duration between 0 and *t*_out_^*u*^; that is, (8) holds:

(8) $$\begin{array}{c} {\text{DA}}_{\left[{0,t}_{\text{out}}^{u}\right]}=100\%. \end{array}$$

*t*_*on*⁡_ denotes the time duration of a friend being online continuously. *f*_*on*⁡_(*t*_*on*⁡_) denotes the pdf of *t*_*on*⁡_. DA of the data published by the user under the given value of *h*, denoted by DA(*h*), can be calculated by combining (7) and (8) as follows:

(9) DA(h)=∫0hfon⁡(toutu) ·(toutuh·DA[0,toutu]  +h−toutuh·DA[toutu,  h])dtoutu.

*h* = *t*_*on*⁡_ + *t*_*off*⁡_ is also a random variable. *f*_*H*_(*h*) denotes the probability density function of *h*, which can be derived from the probability density functions of *t*_*on*⁡_ and *t*_*off*⁡_ and has also been studied in the literature [26, 37].

Therefore, DA of the data published by the user can be finally calculated using

(10) $$\begin{array}{c} \text{DA}=\overset{\infty }{\underset{0}{\int }}\text{DA}\left(h\right)\cdot f_{H}\left(h\right)dh. \end{array}$$

As can be seen from (9), DA is a function over DA_[*t*_out_^*u*^,  *H*]_, which is in turn a function over DA(*t*_in_^*f*^, *t*_out_^*f*^) (shown in (7)). DA(*t*_in_^*f*^, *t*_out_^*f*^) is the function over *t*_*tl*_ (shown in (5)). As shown in (4), *t*_*tl*_ can be calculated from SS. Therefore, we have now established the function of DA over SS.

## 5. Predicting the Data Availability on the Fly

Using the method presented in Section 4, we can calculate SS required to achieve the desired DA of the data published by the user. Note that SS is the total size of the storage pool contributed by all online friends of the user. The friends log in and out dynamically and therefore the number of online friends varies over time. When the number of online friends decreases, the size of the individual storage pool contributed by each online friend has to be increased in order to maintain the desired DA. The existing work in the literature often assumes that the friends of a user are always capable of contributing sufficient storage capacity for the replicated data published by the user. Consequently, there is little work yet in the literature investigating the impact of the friends' dynamic behaviors (i.e., dynamic login and logout) on DA. However, as we have discussed in the introduction section, it is not always acceptable to assume that the friends are willing and able to contribute unlimited storage capacity in the nowadays OSNs. In this paper, we assume that the maximum storage capacity that each friend is able to contribute is *S*. When the required SS exceeds the total storage capacity contributed by all online friends, the DA will drop. Due to the friends' dynamic behaviors, it is very useful to be able to predict the DA on the fly. This section addresses this issue. Consider Figure 1 again. Assume the current time is *t*. The problem of the on-the-fly prediction of DA is to predict the DA at a future time point *t*′(*t*′ > *t*).

According to the discussions above, the key of predicting DA is to predict the number of online friends. At the current time *t*, we know how many friends are online or offline. We can predict the number of friends who are online at a future time *t*′, if we can predict the following two parameters: (i) how many of the friends who are online at time *t* do not change their states from online to offline before or at *t*′, and (ii) how many of the friends who are offline at time *t* change their states to online before or at *t*′. The methods of predicting the above two parameters are presented in Sections 5.1 and 5.2, respectively.  Section 5.3 combines the results obtained in Sections 5.1 and 5.2 to predict the number of online friends and further predict the DA at time *t*′.

### 5.1. Predicting the Number of the Friends Who Are Online at *t* and Do Not Change to Offline before or at *t*′

Given an online friend *v*_*i*_ at time *t*, we can know the time point at which the friend logged in (i.e., became online), which is denoted by *t*_in_*i*_^*on*⁡^. The probability that friend *v*_*i*_ does not change to offline before *t*′ equals the probability that *v*_*i*_ will only log out after *t*′ (i.e., *v*_*i*_'s logout time, denoted by *t*_out_*i*_^*on*⁡^, is greater than *t*′). The probability, denoted by *p*_out_*i*_^*on*⁡^(*t*_out_*i*_^*on*⁡^ > *t*′), in turn equals the probability that *v*_*i*_'s online duration is greater than (*t*′ − *t*_in_*i*_^*on*⁡^) under the condition that *v*_*i*_'s online duration is no less than (*t* − *t*_in_*i*_^*on*⁡^), which can be computed using the conditional probability shown in (11). The condition of (*t*_*on*⁡_ ≥ *t* − *t*_in_*i*_^*on*⁡^) in (11) reflects the fact that *v*_*i*_ has been staying online for the duration of (*t* − *t*_in_*i*_^*on*⁡^):

(11) pout_ion⁡(tout_ion⁡>t′) =pon⁡((ton⁡>t′−tin_ion⁡) ∣ (ton⁡≥t−tin_ion⁡)) =pon⁡(t>t′−tin_ion⁡)pon⁡(t>t−tin_ion⁡) =1−Fon⁡(t′−tinion⁡)1−Fon⁡(t−tinion⁡).

*V*_*on*⁡_ and *N*_*on*⁡_ denote the set and the number of all online friends at time *t*, respectively. Then the number of the friends in *V*_*on*⁡_ who are still online at time *t*′ can be predicted using

(12) $$\begin{array}{c} \overset{N_{\mathrm{on}}}{\underset{i=1}{\sum }}p_{\text{out}\_i}^{\mathrm{on}}\left(t_{\text{out}\_i}^{\mathrm{on}}>t^{\prime }\right). \end{array}$$

### 5.2. Predicting the Number of the Friends Who Are Offline at *t* and Change the States to Online before or at *t*′

The method of predicting the number of the friends who are offline at *t* and change the states to online before or at *t*′ is similar to that presented in Section 5.1:

(13) pin_joff⁡(tin_joff⁡≤t′) =poff⁡((toff⁡≤t′−tout_joff⁡) ∣ (toff⁡≥t−tout_joff⁡)) =poff⁡(t−tout_joff⁡≤toff⁡≤t′−tout_joff⁡)poff⁡(toff⁡≥t−tout_joff⁡) =Foff⁡(t′−tout_joff⁡)−Foff⁡(t−tout_joff⁡)1−Foff⁡(t−tout_joff⁡).

Given an offline friend *v*_*j*_ at time *t*, we can know the time when *v*_*j*_ logged off, denoted by *t*_out_*j*_^*off*⁡^. The probability that *v*_*j*_ changes the state to online before or at *t*′ equals the probability that *v*_*j*_'s login time, *t*_in_*j*_^*off*⁡^, is no later than *t*′. The probability, denoted by *p*_in_*j*_^*off*⁡^(*t*_in_*j*_^*off*⁡^ ≤ *t*′), in turn equals the probability that *v*_*j*_'s offline duration is smaller than (*t*′ − *t*_out_*j*_^*off*⁡^) under the condition that *v*_*j*_'s offline duration is no less than (*t* − *t*_out_*j*_^*off*⁡^), which can be calculated using (13).

*V*_*off*⁡_ and *N*_*off*⁡_ denote the set and the number of all offline friends at time *t*, respectively. Then the number of the friends in *V*_*off*⁡_ who change the states to online before or at time *t*′ can be predicted using

(14) $$\begin{array}{c} \overset{N_{\mathrm{off}}}{\underset{j=1}{\sum }}p_{\text{in}\_j}^{\mathrm{off}}\left(t_{\text{in}\_j}^{\mathrm{off}}\leq t^{\prime }\right). \end{array}$$

### 5.3. Predicting the Number of Online Friends and the DA at *t*′

*N*_*on*⁡_(*t*′) denotes the number of online friends at *t*′. *N*_*on*⁡_(*t*′) can be calculated by (15) by combining (12) and (14) as follows:

(15) Non⁡(t′)=∑i=1Non⁡pout_ion⁡(tout_ion⁡>t′)+∑j=1Noff⁡pin_joff⁡(tin_joff⁡≤t′)=∑i=1Non⁡(1−Fon⁡(t′−tin_ion⁡)1−Fon⁡(t−tin_ion⁡)) +∑j=1Noff⁡(Foff⁡(t′−tout_joff⁡)−Foff⁡(t−tout_joff⁡)1−Foff⁡(t−tout_joff⁡)).

*S* is the maximum storage capacity that each friend is able to contribute. Then the total storage capacity contributed by all online friends at time *t*′ is (*S* · *N*_*on*⁡_(*t*′)). Using the method presented in Section 4, the DA at *t*′ can be determined.

## 6. Case Study 

When we derive the DA model over storage capacity and the on-the-fly prediction of DA in Sections 4 and 5, we used the generic form of the probability distribution for online and offline durations (i.e., *f*_*on*⁡_(*t*_*on*⁡_) and *f*_*off*⁡_(*t*_*off*⁡_)) as well as for the data publishing pattern, that is, the number of times that the user publishes the data in a given time duration (i.e., *P*_pu_(*x*, *t*)). However, it has been shown that the online and offline durations may follow the power-law distribution or the exponential distribution [35, 37, 38] and that the data publishing pattern may follow the Poisson process [37]. In this section, we conduct a few case studies by substituting the generic form of the probability distribution for the power-law, the exponential, and the Poisson distribution. In fact, any probability distributions can be used in the proposed models. Even if the mathematical derivations may not be carried out with some probability distributions, the* Mathematica* software [39] can be used to calculate the model results.

### 6.1. Poisson Distribution

The data publishing pattern may follow the Poisson process [35]. If *X*(*t*_pu_) follows the Poisson distribution with the parameter *λ*_pu_, then we have (16). Consequently, *E*[*X*(*t*_pu_)] can be calculated using (17), as follows:

(16) $$\begin{array}{c} P_{\text{pu}}\left(x\left(t_{\text{pu}}\right)\right)=e^{-\lambda_{\text{pu}}t_{\text{pu}}}\frac{{\left(\lambda_{\text{pu}}t_{\text{pu}}\right)}^{x}}{x!}, \end{array}$$

(17) $$\begin{array}{c} E\left[X\left(t_{\text{pu}}\right)\right]=\lambda_{\text{pu}}t_{\text{pu}}. \end{array}$$

Further, (3) can be transformed to

(18) $$\begin{array}{c} E\left[S\left(t_{\text{pu}}\right)\right]=a\cdot E\left[X\left(t_{\text{pu}}\right)\right]=a\lambda_{\text{pu}}t_{\text{pu}}. \end{array}$$

With (18), (4) becomes

(19) $$\begin{array}{c} ak\lambda_{\text{pu}}\left(t_{\text{out}}^{u}-t_{tl}\right)=\text{SS}. \end{array}$$

Therefore, given the storage capacity SS, the replication degree *k*, and the logout time of the user *t*_out_^*u*^, the publishing time of the oldest data stored in the storage pool, *t*_*tl*_, can be calculated using

(20) $$\begin{array}{c} t_{tl}=t_{\text{out}}^{u}-\frac{\text{SS}}{ak\lambda_{\text{pu}}}. \end{array}$$

Moreover, with (18), (5) then becomes

(21) $$\begin{array}{c} \text{DA}\left(t_{\text{in}}^{f},t_{\text{out}}^{f}\right)=\{\begin{array}{cc} 100\% & t_{\text{out}}^{f}\geq t_{tl}\\ \frac{t_{\text{out}}^{u}-t_{tl}}{{t_{\text{out}}^{u}-t}_{\text{out}}^{f}}\cdot 100\% & t_{\text{out}}^{f}<t_{tl}. \end{array} \end{array}$$

### 6.2. Power-Law Distribution

If the offline duration, *t*_*off*⁡_, follows the power-law distribution with parameter *λ*_*off*⁡_, then we have (22), where *c* = (*λ*_*off*⁡_ − 1)*t*_min⁡_^*λ*_*off*⁡_−1^ given the minimal duration *t*_min⁡_ [40]:

(22) $$\begin{array}{c} f_{\mathrm{off}}\left(t_{\mathrm{off}}\right)=c\cdot {t_{\mathrm{off}}}^{-\lambda_{\mathrm{off}}}. \end{array}$$

We now show how to use the power-law distribution to derive the on-the-fly prediction for the number of online friends, which is obtained in Section 5 through (11), (13), and (15).

Equation (11) can be further derived with the power-law distribution to obtain

(23) pout_ion⁡(tout_ion⁡>t′)pl=1−Fon⁡(t′−tin_ion⁡)1−Fon⁡(t−tin_ion⁡)=1−∫tmin⁡t′−tin_ion⁡cton⁡−λon⁡dton⁡1−∫tmin⁡t−tin_ion⁡cton⁡−λon⁡dton⁡=(t′−tinion⁡t−tinion⁡)1−λon⁡.

Equation (13) can be further derived to obtain

(24) pin_joff⁡(tin_joff⁡≤t′)pl =Foff⁡(t′−tout_joff⁡)−Foff⁡(t−tout_joff⁡)1−Foff⁡(t−tout_joff⁡) =∫t−tout_joff⁡t′−tout_joff⁡ctoff⁡−λoff⁡dtoff⁡1−∫tmin⁡t−tout_joff⁡ctoff⁡−λoff⁡dtoff⁡ =tmin⁡λoff⁡−1((t−tout_joff⁡)1−λoff⁡−(t′−tout_joff⁡)1−λoff⁡)1−tmin⁡λoff⁡−1(tmin⁡1−λoff⁡−(t−tout_joff⁡)1−λoff⁡) =1−(t′−toutjoff⁡t−toutjoff⁡)1−λoff⁡.

Equation (15) can be further derived to

(25) Non⁡(t′)pl=∑i=1Non⁡pout_ion⁡(tout_ion⁡>t′)pl +∑j=1Noff⁡pin_joff⁡(tin_joff⁡≤t′)pl=∑i=1Non⁡(t′−tin_ion⁡t−tin_ion⁡)1−λon⁡ +∑j=1Noff⁡(1−(t′−toutjoff⁡t−toutjoff⁡)1−λoff⁡).

### 6.3. Exponential Distribution

If a random variable *t* follows the exponential distribution with parameter *λ*, then its probability density function and probability distribution function can be expressed as in

(26) $$\begin{array}{c} f\left(t\right)=\lambda e^{-\lambda t},\\ F\left(t\right)=1-e^{-\lambda t}. \end{array}$$

We now show how to use the exponential distribution to derive the on-the-fly prediction for the number of online friends.

With the exponential distribution, (11) can be derived to obtain

(27) poution⁡(toution⁡>t′)exp⁡ =1−Fon⁡(t′−tinion⁡)1−Fon⁡(t−tinion⁡) =1−(1−e−λon⁡(t′−tinion⁡))1−(1−e−λon⁡(t−tinion⁡)) =e−λon⁡(t′−t).

Also, (13) can be transformed to

(28) pin_joff⁡(tin_joff⁡≤t′)exp⁡ =Foff⁡(t′−tout_joff⁡)−Foff⁡(t−tout_joff⁡)1−Foff⁡(t−tout_joff⁡) =e−λoff⁡(t−toutjoff⁡)−e−λoff⁡(t′−toutjoff⁡)e−λoff⁡(t−toutjoff⁡) =1−e−λoff⁡·(t′−t).

Further, (15) then becomes

(29) Non⁡(t′)exp⁡=∑i=1Non⁡pout_ion⁡(tout_ion⁡>t′) +∑j=1Noff⁡pin_joff⁡(tin_joff⁡≤t′)=Non⁡·(e−λon⁡·(t′−t))+Noff⁡·(1−e−λoff⁡·(t′−t)).

## 7. Evaluation

A discrete simulator has been developed in this work to simulate an OSN. There are *N* users in the simulated OSN. Some users act as the friends of another user and update the data published by the user. The online and offline durations of the users in the simulated OSN follow the power-law distribution (PL) or the exponential distribution (Exp), as observed in the literature [37]. The user publishes the data following the Poisson process and *k* copies of replicas are created for each data item and stored in the online friends.

In order to evaluate the DA model over storage capacity, the DA is predicted given the size of storage capacity and the values of other OSN parameters. Then the simulated OSN is run using those parameters values. Each friend contributes the same storage capacity and the storage capacity is allowed to be adjusted so that the total storage capacity of all online friends always equals the storage capacity used to predict the DA. During the running, when a friend comes online at a time point, the DA of the published data for the friend is recorded. The average of all recorded DA is regarded as the actual DA, which is compared against the predicted DA to measure the accuracy of the prediction.

**Figure 2:**
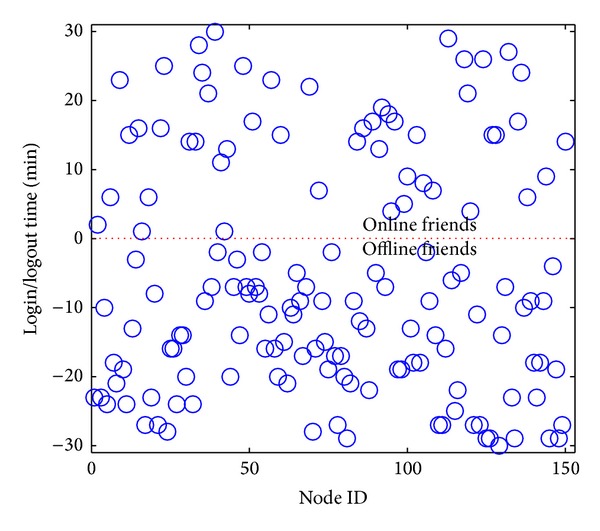
The states of all friends at current time point.

In order to evaluate the on-the-fly prediction, the experimental scenario is designed as follows. A user and his friends log in and out following the specified distribution during the time interval [0, *l*]. The current time is set to be *m*th min (*m* < *l* and the user is offline at time *m*). The online or offline states of all friends at time *m* as well as the latest login or logout time before time *m* are collected. The collected data, combining with the specified distributions, are used to predict the number of online friends and DA at the future time points (i.e., the time points later than *m*). The predicted data are then compared against the data obtained from the actual running. For example, the number of the friends of a user is set to be 150.  Figure 2 shows the online/offline state of each friend when the current time is set to be 31st min. A point above the red line (i.e., when *y* = 0) represents the latest login time of a friend who is online at 31st min, while a point below the red line shows the latest logout time of a friend who is offline at 31st min.

In the rest of this section, the DA model over storage capacity is evaluated in Section 7.1 with regard to the following aspects: (i) the impact of storage capacity on DA, (ii) the impact of the DOSN parameters, including online/offline duration and the rate of user publishing data, on DA, and (iii) the accuracy of the relation established between DA and SS.

In Section 7.2, the on-the-fly prediction is evaluated with regard to the following aspects: (i) the accuracy of predicting the number of online friends on the fly and (ii) the accuracy of the DA predicted on the fly.

Unless stated otherwise, the experimental parameters used in the performance evaluations take the values shown in Table 2. These values are chosen based on those used in the literature [19, 35, 37].

### 7.1. Evaluating the DA Model over Storage Capacity

#### 7.1.1. Impact of Storage Capacity on DA

**Figure 3:**
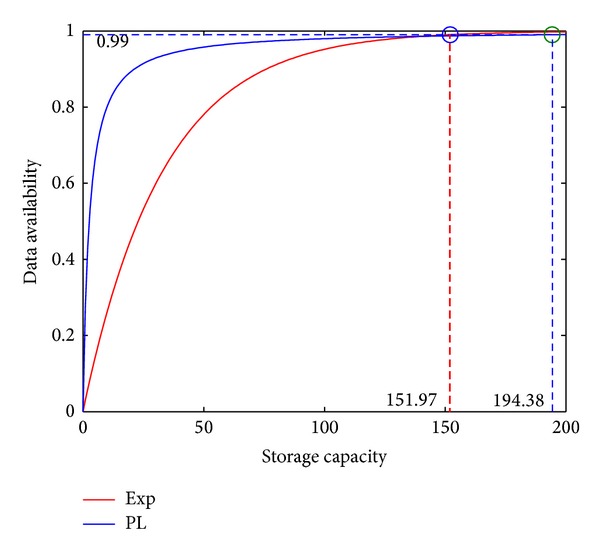
The impact of SS on DA.

Figure 3 shows the impact of the total storage capacity (i.e., SS in Section 4) on the DA calculated from the DA model presented in Section 4. As shown in Figure 3, the DA increases as SS increases. Under both exponential distribution and power-law distribution of the friends' online duration, data availability tails off after SS increases more than a certain value. These results suggest that it is unnecessary to ask the friends to contribute unlimited storage capacity, as often assumed in the work in the literature [14, 18].

From this figure, we can also determine SS that is required to achieve a certain DA. For example, DA reaches 99% under PL or Exp when SS is 194.38 and 151.97, respectively.

#### 7.1.2. Impact of On/Offline Durations on DA

As can be seen from the derivation of the DA model presented in Section 4, the online/offline durations have impact on DA. We conducted the experiments to evaluate their impact. Since the online and offline durations have the similar impact, only the results for offline durations are presented in this subsection. Given the distribution of the offline duration, the average duration is controlled by *λ*_*off*⁡_. The inverse of *λ*_*off*⁡_ is the length of the duration.

**Figure 4:**
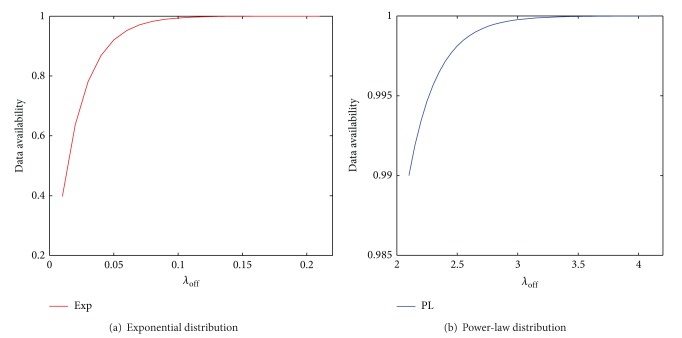
The impact of the offline durations on DA.

Figure 4 shows the impact of *λ*_*off*⁡_ on DA. In the experiments in Figure 4, SS is set to be 194.38 and 151.97 under PL and Exp (as shown in Figure 3), respectively, so that DA is 99% under the default value of *λ*_*off*⁡_ (as in Table 2). We then change the value of *λ*_*off*⁡_ and plot the corresponding DA. It can be observed that DA increases as *λ*_*off*⁡_ increases under both Exp and PL. These results can be explained as follows. When *λ*_*off*⁡_ increases, the average length of the friends' offline durations decreases. Given the certain SS, the period of the stored data (i.e., [*t*_*tl*_, *t*]) is fixed. Therefore, the shorter offline durations of the friends result in higher probability that the times of the data that the friends try to update fall into [*t*_*tl*_, *t*]. Consequently, DA is higher.

#### 7.1.3. Impact of the Data Publishing Rate on DA

From the DA model, we can also know that the pattern with which the user publishes data has the impact on DA. It is shown in the literature that the number of times that the user publishes the data in a duration follows the Poisson distribution. Then, the parameter of the Poisson distribution, *λ*_pu_, reflects the data publishing rate. The higher the *λ*_pu_, the higher the data publishing rate.

**Figure 5:**
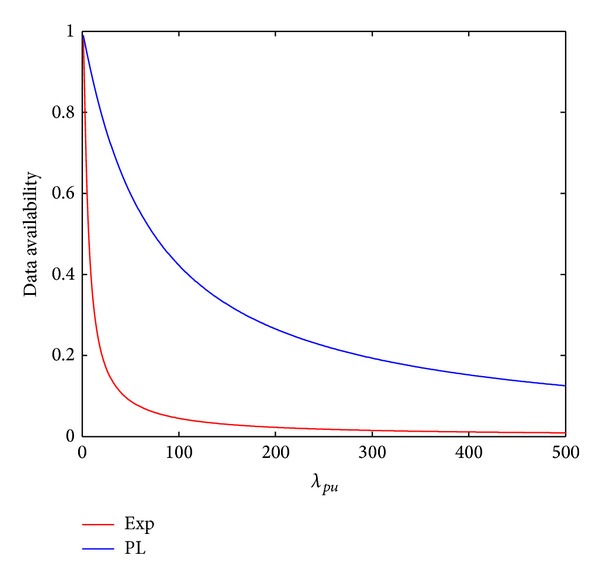
The impact of the data publishing rate on DA.

Figure 5 demonstrates the impact of *λ*_pu_ on DA. The setting of SS is the same as that in Figure 4. The figure shows that DA decreases as *λ*_pu_ increases. This is because when the data are published at a higher rate, [*t*_*tl*_, *t*] is shorter given a fixed SS. Consequently, DA is lower.

#### 7.1.4. Accuracy of the DA Model

**Figure 6:**
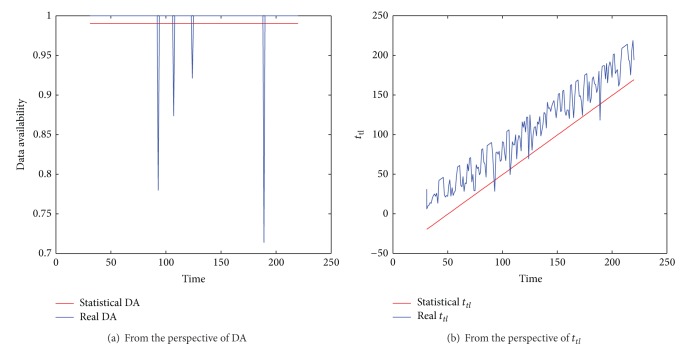
The accuracy of the DA model.

The DA model over storage capacity proposed in Section 4 can calculate the DA given an SS. We conducted the experiments to study how accurate the calculated DA is, compared with the DA obtained from the actual running. The results are presented in Figure 6. The results under Exp and PL show similar pattern. Therefore, only the results under Exp are presented.

In Figure 6, the setting of SS is the same as that in Figure 4 (i.e., SS = 151.97). The DA calculated by the DA model is 99%, which is the red line in Figure 6(a). We run the simulated OSN with this SS and plot the actual DA over time, which is the blue line in Figure 6(a). It can be seen that the DA is fairly close to the calculated DA in most cases. These results suggest that the DA model is effective. In order to reveal the fundamental reason for this, we also compared *t*_*tl*_ obtained in the DA model (the red line in Figure 6(b)) with the time of the oldest data that a friend tried to update when he came online at a time point (plotted in blue in Figure 6(b)). If the time of the oldest data is not earlier than the calculated *t*_*tl*_, the DA model is effective. As can be seen from Figure 6(b), the blue line is higher (i.e., the corresponding time is later) than the red line in most cases. This gives the fundamental reason why the DA model is effective; that is, with the SS obtained by the DA model, the online friends can in most cases store the data that a friend tries to update when he comes online.

### 7.2. Evaluating the on-the-Fly Prediction of DA

#### 7.2.1. Accuracy of the Predicted Number of Online Friends and the Impact of Online and Offline Durations

**Figure 7:**
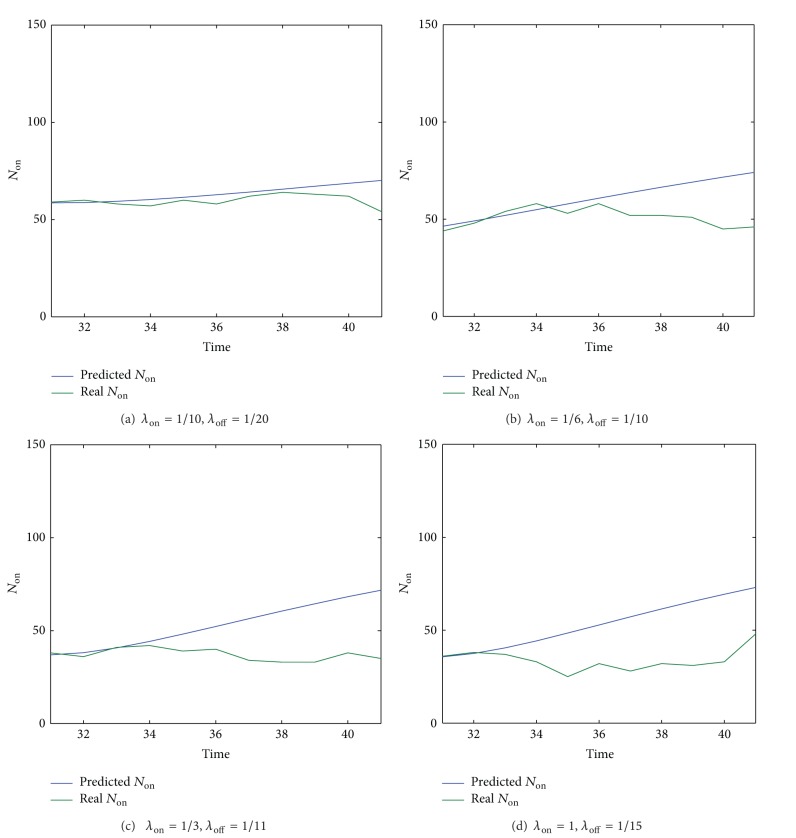
The accuracy of prediction model over time.

As shown in Section 5, the predicted number of online friends (i.e., *N*_*on*⁡_) determines the value of the on-the-fly DA. Therefore, we conducted the experiments to evaluate the accuracy of predicting *N*_*on*⁡_. The experimental scenario has been presented in the third paragraph of Section 7. The experimental results are shown in Figure 7. In Figure 7, the current time point is set to be 31st min and the on-the-fly prediction predicts *N*_*on*⁡_ from 31st min onwards, which is plotted in blue. The actual *N*_*on*⁡_ from 31st min onwards is plotted in green. Figures 7(a), 7(b), 7(c), and 7(d) show the results under different *λ*_*on*⁡_ and *λ*_*off*⁡_ (i.e., online and offline durations).

It can be seen from Figure 7(a) that, compared with its actual values, the prediction of *N*_*on*⁡_ is fairly accurate in the first 10 minutes, which shows the effectiveness and applicability of the proposed prediction method since the prediction can be conducted on the fly as the time elapses. By comparing Figures 7(a), 7(b), 7(c), and 7(d), we can see that the length of the accurate prediction decreases as the settings of *λ*_*on*⁡_ and *λ*_*off*⁡_ change from Figures 7(a)–7(d). These results indicate that the online and offline durations have impact on the prediction accuracy. After carefully analyzing the changing trend of *λ*_*on*⁡_ and *λ*_*off*⁡_, it appears that the minimum value between the online and the offline durations (i.e., min(1/*λ*_*on*⁡_, 1/*λ*_*off*⁡_)) determines the length of accurate prediction. The less the value of min(1/*λ*_*on*⁡_, 1/*λ*_*off*⁡_), the shorter the length of the accurate prediction. The reason for this is because when min(1/*λ*_*on*⁡_, 1/*λ*_*off*⁡_) is smaller, the friends are more dynamic and, consequently, it is more difficult to obtain the accurate prediction in the future.

#### 7.2.2. Accuracy of the Predicted DA

**Figure 8:**
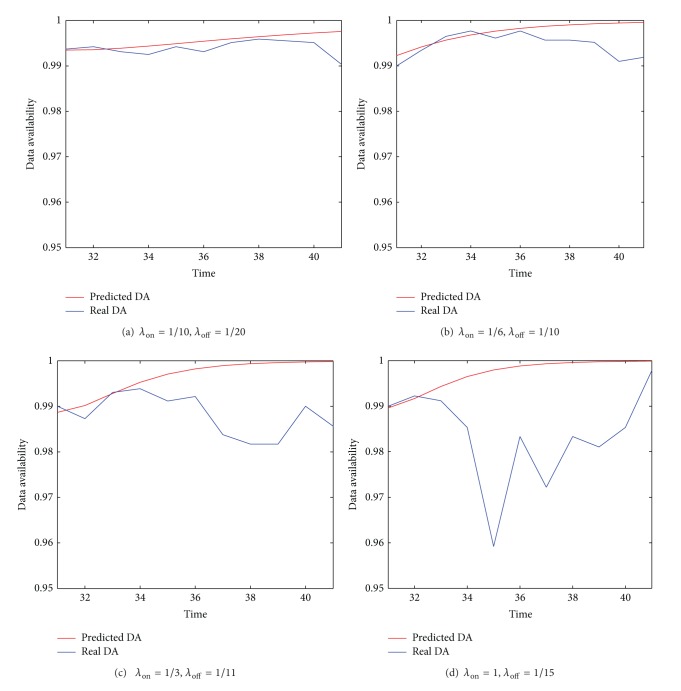
The accuracy of the on-the-fly prediction of DA.

Finally, Figure 8 presents the experiments results that show the accuracy of the on-the-fly prediction of DA. The experimental settings in Figure 8 are the same as those in Figure 7. It can be seen from Figure 8 that the trends shown in Figure 8 are consistent with those in Figure 7. This once again shows the effectiveness of the on-the-fly prediction.

## 8. Conclusions

This paper proposes a data availability model over storage capacity for DOSNs. Further, a novel method is proposed to predict the data availability on the fly. Extensive simulation experiments have been conducted. The results show that the proposed data availability method is able to capture the relation between data availability and storage capacity effectively, and that the on-the-fly prediction method can predict the level of data availability accurately.

This work is situated at the level of maintaining the data availability. How to optimize the data accessing performance and reduce the data maintenance overhead is the work of the underlying data replication and placement strategies. In the future, we plan to work down the management level in DOSN and develop the strategies of placing data replicas among friends in DOSN. When designing the placement strategies, the attributes of individual friends, such as the bandwidth and latency associated with a friend and the storage capacity contributed by a friend, will be taken into account.

## Figures and Tables

**Table 1:** The notations that are used in the derivation.

Notations	Descriptions
*v*_*t*_	The user

*N*	The number of the user's friends

*t*	Current time point

*t*′	Target time point in near future, *t*′ = *t* + Δ*t*, where Δ*t* is a time duration after *t*. We want to predict the state of the DOSN at the time point *t*′

*t*_out_^*u*^	The time point at which the user *v*_*t*_ goes offline

*V*_*on*⁡_	The set of all online users in the friend circle of the user *v*_*t*_ at current time *t*

*N*_*on*⁡_	The number of online users in set *V*_*on*⁡_

*V*_*off*⁡_	The set of all offline users in the friend circle of the user *v*_*t*_ at current time *t*

*N*_*off*⁡_	The number of offline users in set *V*_*on*⁡_

*t*_in_*i*_^*on*⁡^	The latest login time of the online user *v*_*i*_ in *V*_*on*⁡_ before current time *t*

*t*_out_*i*_^*on*⁡^	The first logout time of the online user *v*_*i*_ in *V*_*on*⁡_ after current time *t*

*t*_out_*j*_^*off*⁡^	The latest logout time of the offline user *v*_*j*_ in *V*_*off*⁡_ before current time *t*

*t*_in_*j*_^*off*⁡^	The first login time of the offline user *v*_*j*_ in *V*_*off*⁡_ after current time *t*

*E*_login_ *E*_logout_	The login and logout events, respectively. When any of these two events occurs, the state of a user changes from *OFFLINE* to *ONLINE* or from *ONLINE* to *OFFLINE *

*t*_*on*⁡_ *f*_*on*⁡_(*t*_*on*⁡_) *F*_*on*⁡_(*t*_*on*⁡_)	The time duration of a user being online continuously (i.e., the time duration from an *E*_login_ event to the following *E*_logout_ event), which is a random variable and whose probability density function and probability distribution function are denoted by *f*_*on*⁡_(*t*_*on*⁡_) and *F*_*on*⁡_(*t*_*on*⁡_), respectively

*t*_*off*⁡_ *f*_*off*⁡_(*t*_*off*⁡_) *F*_*off*⁡_(*t*_*off*⁡_)	The time duration of a user being offline, which is also a random variable and whose probability density function and probability distribution function are denoted by *f*_*off*⁡_(*t*_*off*⁡_) and *F*_*off*⁡_(*t*_*off*⁡_), respectively

*x* *P*_pu_(*x*, *t*)	The number of times that the user publishes the data, which is a discrete random variable and whose probability density function in a duration *t *is denoted by *P*_pu_(*x*, *t*)

*a*	The statistical average size of the data published by the user each time. *a* is a constant

*k*	The replication degree, that is, the number of replicas created for each data item

*t*_*tl*_	The publishing time of the oldest data stored in the storage pool

SS	The total storage capacity contributed by all online friends

*S *	The maximum storage capacity that each friend is able to contribute

**Table 2:** The default parameters in performance evaluations.

Notations	Default value	Descriptions
*N*	150	The number of the user's friends
*a *	1	The average size of published data
*λ*_*on*⁡_^exp⁡^	1/3	The parameter of the online time duration which follows exponential distribution
*λ*_*off*⁡_^exp⁡^	1/11	The parameter of the offline time duration which follows exponential distribution
*λ*_*on*⁡_^pl^	2.5	The parameter of the online time duration which follows power-law distribution
*λ*_*off*⁡_^pl^	2.1	The parameter of the offline time duration which follows power-law distribution
*λ*_pu_^ps^	1	The parameter of the number of times the user publishes data which follows Poisson distribution
